# Remarks Concerning the Hygiene of Military Hospitals, Extracted from a Paper Read before the Imperial Academy of Medicine at Paris

**Published:** 1864-03

**Authors:** M. H. Baron Larrey, J. L. Cabell

**Affiliations:** Paris, 1861; University of Virginia, Surgeon P. A. C. S.


### CHRONICLE OF MEDICAL SCIENCE.

ART. I.—*Remarks concerning the Hygiene of Military Hospitals, extracted from a paper read before the Imperial Academy of Medicine at Paris, by* M. H. BARON LARREY. Paris, 1862. [Translated from the French and abridged by Professor J. L. CABELL, University of Virginia, Surgeon P. A. C. S.] (Concluded.)

The author next alludes to disinfectants, recommended as a means of remedying, in some measure, the defects of ventilation, and gives the preference to permanent fumigations of chloride of lime diluted with water, by means of vessels scattered through the wards and passages, the chloride being removed every three or four days.

"A substantial, nourishing diet exerts the happiest influence on the cure of patients exhausted by hemorrhages, by suppuration, by abstinence, and by protracted confinement in bed. It is this, in combination with efficient ventilation, which contributes to the excellent sanitary condition of the hospitals in London, where the subjects of surgical operations are treated from the first day according to the English regimen—wine, boiled beef, and opium in fractional doses. This regimen, omitting the opium, furnishes good results in the country and in field hospitals against debilitating diseases, and particularly against scurvy."

[I have obtained excellent results in cases of protracted suppuration connected with compound gun-shot fractures of the extremities by the use of *malt liquors*, when distilled spirits had altogether failed, and had even proved hurtful. They promote sleep, they are directly nutritious, and they increase the appetite and the power of digestion. If their manufacture were encouraged, very much of the alcoholic stimulus now uselessly given to hospital patients might be saved.—J. L. C.]

Daily exercise by convalescents produces a double advantage for themselves and for the sick, who are benefitted by their absence from the ward.

A premature discharge of the convalescents from hospitals exposes them to relapses or other accidents which may yet be more grave than the primary disease.

It is only by sending such convalescents to other places that the hospital, too, can be preserved from the dangers of over-crowding.

The great mortality of military hospitals is a vast and complex question deserving the most serious attention. I do not now propose to speak of the excess of mortality due to the military profession, the subject of an interesting work by M. Tholozan, but there are other conditions inherent in the hospital system. "It is incontestible," says M. Michael Levy, "that the mortality is proportionally greater in the large than the small hospitals. We can never congregate many thousand sick men into one establishment with impunity. From 1,000 to 1,200 is a maximum beyond which it becomes very difficult to avoid the danger of infection." We may note also insufficient means of hygiene, defect of aëration, imperfect quality of food, and other influences already incidentally mentioned, as so many causes of a high mortality. "We must add to these causes the direct effect of a great number of the major surgical operations." [More likely to be followed by dangerous and mortal complications, as pyæmia, sloughing, phagedænia, &c., in hospitals than in private quarters.]

But the predominant cause of hospital mortality will always be found to depend on the influences resulting from crowding a number of men into one building.

Among the numerous facts which demonstrate this proposition I will cite but one at present. It belongs to the history of Paris in 1814. The ratio of mortality in the military hospital of Val-de-Grace at Paris was increased ten fold as the consequence of excessive crowding on the occasion of the return of the army, together with the foreign troops. At length the proper authorities provided for regular evacuations and for all other hygienic measures having for their object to diminish the crowd, and the excess of mortality did not re-appear. On that occasion the great abattous, [establishments in Paris for the butchering of cattle for the markets,] transformed into temporary hospitals, exhibited the most satisfactory results.

All the documents which we possess on the medico-chirurgical history of the Crimean campaign; the special publications of the numerous French and English writers; the statistical tables drawn up with the greatest care, for the French army, by M. Chener; and especially the unpublished official reports of inspectors Michael Levy and Baudens—all bear witness, from different points of view, to the fatal effects of over-crowding in the ambulance hospitals and in hospitals proper. M. Michael Levy signalized the formidable congregation of 2,100 beds in the Hospital at Pera, and the ravages occasioned at Varna by epidemic cholera and hospital gangrene. He demonstrates that all hospitals having more than 800 beds become infected, and that the accumulation of the wounded, of patients who have undergone operations, those suffering from dysentery, and the subjects of scurvy, may engender contagious and fatal diseases. He cites the case of an over-crowded ship having 1,200 men on board, of whom 350 were attacked with cholera, and 72 died in forty-eight hours.

Inspector Baudens clearly proves that the collection of too large a number of patients in a hospital is a fruitful cause of grave fevers, dysentery, purulent resorption, hospital gangrene, and of typhus. He substantiated the existence of such consequences, both in the Crimea and at Constantinople, and persistently recommended the most rational hygienic measures; the thinning out of the beds; the disinfection of the wards, and the discharge of the sick; regretting that the medical and administrative authorities could not appreciate the full force and import of the word "over-crowding."

Numerous typhoidal diseases appeared towards the close of the year 1855, and multiplied rapidly among the French and English troops. They were much more aggravated during the more rigorous winter of 1856, among the French and Piedmontese, by the over-crowding of camps, the ambulance hospitals and hospitals proper, which they sensibly diminished, and then disappeared in the English army, under the influence of a better system of hygiene and despite the infectious emanations of the neighboring cemeteries. Results entirely analogous were observed in the hospitals of Constantinople, and sometimes in the same hospital, in proportion as the crowding, the immediate cause of typhus, increased or diminished. Thus the French hospital of Rami Tehifflich, one of the most favorably situated in the whole city, remained in a satisfactory sanitary condition by the limitation of the number of beds to 900, under M. Cazaler, and subsequently under his successor, M. Ganeau. But, at a later epoch, another physician of great respectability and merit, having less fears of the consequences of over-crowding the sick and trusting in the material resources at his command, increased the number of beds to 1,200 and then to 1,400. The hospital, from that time kept filled to its limits, soon became a focus of typhus fever, and a frightful mortality ensued, which spared neither the attendants nor the medical officers, of whom the officer in charge, who had over-crowded the hospitals, was one of the first victims. M. Cavillon, who succeeded him, was soon attacked with this fatal epidemic, and barely escaped with his life in losing his reason.

The military hospitals at Pera, nearly as much over-crowded as that at Rami-Tehifflich, suffered in a precisely similar way.

M. F. Jacquot estimated the mortality from typhus fever in all the hospitals at Constantinople as fully one-half of the entire mortality.

On the other hand, in one of the hospitals which has never been over-crowded, the number of deaths from typhus was only one-tenth of the entire mortality, which, too, was lower than in preceding years at the same epoch. In like manner, the civil hospital at Pera, perfectly organized for 90 beds, and having received, between the 1st of January and 30th of April, 300 sick, including 100 cases of typhus, had not a single case of typhus developed in its wards. Of the 100 received, 15 only died.

Results not less conclusive were observed in the ambulances [field hospitals] in the Crimea. Intended for from 200 to 400 men each, they were sometimes filled to more than double that number. Thus a field hospital of the first division of the third corps, 15 out of 16 medical officers were taken sick. In other field hospitals, better arranged and especially not crowded, typhoidal affections were exceptional cases. Finally, in field hospitals, which were invaded by typhus, the progress of the disease was always arrested by increasing the number of tents and widening the intervals between them.

The mortality in the French army, during the entire Crimean campaign, was 67,056 out of 309,268 troops, more than half being due to disease. Of 160 Sisters of Charity employed in the hospitals at Constantinople, 68 had typhus fever in 1856; 14 were treated in more or less crowded hospitals, and 11 died; whilst of the other 54, transferred to an infirmary at Galata, the hygienic conditions of which, especially the absence of over-crowding, were perfect and satisfactory, not one proved fatal.

The author of this report being ordinary surgeon of the French Emperor, and having been appointed chief surgeon of the French army for the Italian campaign in 1859, carried out the principles deduced from the experience of the Crimean war and was rewarded by results of the most gratifying character. He ascribes these results to the persistent "application of one radical measure, namely the distribution of the wounded in numerous small hospitals. No matter how small, in preference to packing or crowding a large number of men into a few buildings, however large." It is to be remembered that this policy of scattering the sick is completed by a measure necessary to it, that, namely, of occasionally removing the sick and wounded from one locality to another.

One capital fact must be signalized as an irrefragable proof of the beneficial influence of the sanitary measures put in practice in Italy, and it is said that there were no epidemics, properly speaking, during the whole of that campaign.

*Conclusions.*

The following conclusions are deduced from the foregoing statement:

The multiplied and complex influences of the vitiation of the atmosphere of hospitals proceed from over-crowding and determine the most disastrous effects. This proposition cannot be too frequently reiterated, if we would avoid the error of substituting secondary questions for the fundamental one.

All partial measures are demonstrably insufficient. The suppression or the closure of certain hospitals, presumed to be insalubrious, would be useless if it could be tried. Neither would it suffice to modify the larger buildings in order to assimilate them to the small proportions of others. But the necessity of improving all by multiplying their number, in order to reduce the number of beds in each one, and to insure a wide dissemination of the sick and wounded to such a degree that, when epidemics prevail, it would be better to close certain hospitals than to fill them—the necessity of a generous nourishment and a regular system of supervision, with respect to the enforcement of the rules of hospital hygiene, such are the ends at which we should aim.

CONFEDERATE STATES

